# Neuroinflammation: A Driving Force in the Onset and Progression of Alzheimer’s Disease

**DOI:** 10.3390/jcm14020331

**Published:** 2025-01-08

**Authors:** Campbell Long, Arianne Fritts, Jessica Broadway, Olga Brawman-Mintzer, Jacobo Mintzer

**Affiliations:** 1 Ralph H. Johnson VA Health Care System, 109 Bee St, Charleston, SC 29401, USA; 2 College of Health Professions, Department of Health Sciences and Research, Medical University of South Carolina, 171 Ashley Avenue, Charleston, SC 29425, USA; 3 College of Medicine, Department of Psychiatry and Behavioral Sciences, Medical University of South Carolina, 171 Ashley Avenue, Charleston, SC 29425, USA; 4 College of Health Professions, Department of Health Studies, Medical University of South Carolina, 171 Ashley Avenue, Charleston, SC 29425, USA

**Keywords:** Alzheimer’s disease, amyloid, neuroinflammation, peripheral inflammation, microglia, cytokines, treatment

## Abstract

**Background/Objectives**: The goal of this commentary is to highlight several key components of the inflammatory process as it relates to amyloid toxicity in Alzheimer’s disease (AD), including the role of neuroinflammatory factors and peripheral inflammatory events. **Methods**: Google Scholar and PubMed were used to find articles with the following keywords: Alzheimer’s disease, amyloids, neuroinflammation, peripheral inflammation, microglia, cytokines, and treatments. Sources that were case reports, not peer-reviewed, or older than 30 years were excluded. Abstracts were reviewed first for their relevance before the full text was considered. Methods sections were reviewed to ensure the interventional papers included were randomized controlled trials, meta-analyses, or systematic reviews; however, several literature reviews were also included due to the relevance of their background information. **Results**: Based on the literature review, we chose to concentrate on microglia, cytokine signaling, and peripheral inflammation markers. We found that microglia activation and subsequent microglia-driven inflammation play a pivotal role in the pathomechanism of AD. Additionally, cytokines such as interleukin-6 (IL-6) and tumor necrosis factor-alpha (TNF-a) appear to contribute to amyloid accumulation and cell damage. Finally, the increased permeability of the blood–brain barrier (BBB) allows for the peripheral inflammatory process to contribute to the inflammation of the central nervous system (CNS) and amyloid-beta (Aβ) accumulation. **Conclusions**: Current evidence suggests that the immune system plays a pivotal role in the pathogenesis of AD, both in the CNS and the periphery.

## 1. Introduction

Alzheimer’s disease (AD), the most common age-associated neurodegenerative disease, is estimated to affect over 50 million people worldwide. In the US alone, an estimated USD 321+ billion is spent on the direct care of patients with AD each year [1]. As the nation’s population continues to age, this number is only expected to increase. AD is characterized by several hallmark traits, including the deposition of beta amyloids (Aβ) and their subsequent accumulation into senile plaque, as well as the hyperphosphorylation of the tau protein that results in the structural dysfunction of neurons and their eventual collapse into neurofibrillary tangles [2]. In recent years, however, research has begun to examine a third hallmark of AD: inflammation. Emerging studies are shedding light on how inflammatory responses in the central nervous system (CNS) and peripheral nervous system (PNS) act as both risk and maintenance factors for AD. The goal of this commentary was not to perform an in-depth and systematic review of the literature but rather to aid clinicians in the understanding of key emerging themes as they relate to immune function and AD pathology. This review highlights several key components of inflammation as it relates to amyloid toxicity in AD, including the role of neuroinflammatory factors as well as peripheral inflammatory events.

## 2. Methods

We conducted research using Google Scholar and PubMed to identify relevant articles. The literature search pulled papers from 1 January 1996 to 29 June 2024. We utilized the following keywords: Alzheimer’s disease AND amyloid AND neuroinflammation OR peripheral inflammation AND microglia AND cytokines AND treatments. Sources that were case reports, not peer-reviewed, or outside the range of 1996–2024 were excluded from the review. Seventy articles were identified. Abstracts were reviewed first for their relevance before the full text was considered. Articles were then more thoroughly evaluated by CBL and JEM, including an assessment of the Methods sections to ensure the quality of data collection. In general, the papers included were randomized controlled trials, meta-analyses, or systematic reviews; however, several literature reviews were also included due to the relevance of their background information.

## 3. Results

### 3.1. Microglia

Until recently, the role of the innate immune response in AD pathology has remained poorly characterized. However, scientists have begun to examine the immunologic mechanisms relevant to both the onset and progression of this degenerative disease. Among the most important emerging evidence is the role of microglia in both the deposition and clearance of amyloids within the brain parenchyma, as well as the inflammation resulting from their prolonged activation.

Microglia are the resident immune cells of the brain, and they contribute to maintaining CNS homeostasis through both neurogenesis and phagocytic processes. Traditionally, microglia have been classified by the M1/M2 phenotypic paradigm, which divides macrophages into M1, known classically as the key components of inflammatory response, and M2, known as wound-healing macrophages. But new information regarding their behavior in vivo renders this classification insufficient. Microglia are dynamic, with a continuum of behavior that is dependent on situational factors [3]. In the absence of local or peripheral insults, microglia can exert neurotrophic effects through the release of anti-inflammatory cytokines, growth factors, and synaptic pruning. However, threats to homeostasis, such as danger-associated molecular patterns (DAMPs) like Aβ, trigger a fundamental change in microglial processes via the innate immune response. These so-called “activated” microglia are capable of inducing neuroinflammation through wide-scale microglial recruitment and phenotypic changes to their structure. Swollen, amoebic-like cell bodies become more prevalent and are capable of releasing proinflammatory chemokines and cytokines, as well as generating excitotoxic compounds, such as reactive oxygen species (ROS), which can contribute to disease progression [4]. However, the evidence shows that if microglia successfully eliminate the threat, these cells are capable of returning to an “inactivated” form via anti-inflammatory cytokine pathway signaling [5]. For example, triggering receptors expressed on myeloid cell 2 (TREM2) signaling aids in the microglia’s return to a non-activated state. Researchers found that increased soluble TREM2 in the CSF is associated with decreased amyloid uptake on PET imaging, but decreased soluble TREM2 correlated with greater amyloid uptake [6]. These results indicate that lower levels of TREM2 in the CSF may be an important early biomarker of microglial dysfunction as it relates to their activated, inflammatory state and subsequent ability to clear amyloids.

In a healthy setting, microglia facilitate the disposal of Aβ oligomers and help maintain homeostasis via the clearance of Aβ deposits downstream into the CSF. Unfortunately, microglia are often not capable of completely ridding the CNS of Aβ. When Aβ plaque accumulates, it is quickly surrounded and penetrated by microglia in an attempt to prevent their outward growth toward nearby neurons. Internalized Aβ protofibrils are delivered to lysosomes within the microglia for destruction and subsequent clearance via lysosomal proteases. However, the insufficient conveyance of chloride transport CIC-7 (a gene that codes for a chloride channel in late endosomes and lysosomes) to the lysosomes results in an incomplete breakdown of Aβ fibrils and, ultimately, a failure of clearance from the affected parenchyma [7]. In turn, more microglia are recruited to the plaque via the recognition of DAMPs in the Aβ itself and subsequent inflammatory cytokine signaling from the existing microglia. A gross influx of activated microglial cells to affected areas has been linked with aberrant synaptic pruning, the increased release of damaging ROS, and increased production of matrix metalloproteinase-3 (MMP-3), which has been shown to degrade the extracellular matrix [8]. In concert, these effects contribute to the hallmark cognitive decline seen in neurodegenerative diseases like AD.

If unbridled microglial activation and subsequent inflammation play such a pivotal role in the pathomechanism of AD, what, if anything, can be put in action to address it? Novel gene therapy compounds targeting the activation of microglia themselves may hold promise for the advancement of treating AD. One such drug of interest, MCC950, potently inhibits the NOD-like receptor family pyrin domain-containing 3 (NLRP3) [9]. NLRP3 is an inflammasome abundantly present in microglia and is responsible for the activation of several proinflammatory signalers, or cytokines, in the interleukin family. MCC950 prevents the actuation of NLRP3 by several immune processes and, thus, inhibits the release of these inflammatory agents, which has been hypothesized to prevent the further proliferation of microglial activation [10]. However, a phase II trial of the MCC950 compound, assessing its effects in rheumatoid arthritis, was prematurely terminated due to indicators of potential liver toxicity [10]. Based on its potential effects on AD animal models, this compound may be of interest as a therapeutic target if careful surveillance of liver function is adopted [9].

Some studies have turned attention to nonsteroidal anti-inflammatory drugs (NSAIDs) as a means of reducing these microglial-driven inflammatory processes. Traditionally, NSAIDs work by inhibiting the cyclooxygenase (COX) enzymes responsible for producing hormone-like structures that cause pain and inflammation [11]. In vitro rodent models involving the fenamate class of NSAIDs (such as mefenamic acid and flufenamic acid) have successfully prevented the release of several proinflammatory signals by macrophage cells, namely by inhibiting NLRP3 independent of COX1 or COX2 inhibition [12]. Moreover, in vivo mouse models demonstrated the protective effect of fenamate NSAIDs against cognitive decline in mice injected with soluble Aβ oligomers, with effects persisting up to 21 days after treatment ceased [12]. These results show promise but will require more trials in the human AD population to confirm their clinical relevance.

In a meta-analysis of 17 epidemiological studies, researchers found that long-term use of NSAIDs (over both a 5- and 10-year period) significantly reduced the risk of developing AD, with longer use associated with the greatest reduction in risk [13]. The type of NSAID was not assessed, however, and these results could not be replicated in more recent prospective trials with clinical AD populations [14]. These null results may be due, in part, to the fact that neuroinflammation begins early in the development of AD, and NSAID intervention at a symptomatic stage in the disease process may be a case of “too little, too late”. If the evidence points to NSAIDs mitigating the risk of developing AD rather than feasible treatment after symptoms appear, then more research may be warranted on their use as a form of preclinical intervention in slowing the progression of microglial-driven neuroinflammation. Admittedly, previous prospective trials in presymptomatic AD populations returned less-than-favorable results, but imperfect screening practices coupled with shorter treatment periods (<3 years) may also account for these negative findings [14]. After all, the majority of studies with positive results examined NSAID treatment periods of 5+ years. Given these facts, more research is needed to elucidate the potentially protective effects of long-term NSAID use in preclinical, asymptomatic populations.

### 3.2. Cytokine Signaling

To understand how microglial activation goes awry, it is important to examine the relevant pathways of intracellular communication, namely the role of cytokine signaling in immune response and its subsequent effects on microglia. Cytokines are signal proteins produced by cells that are essential for intercellular communication and the regulation of the immune system. Because they are too large to cross the lipid bilayer of the cell wall, cytokines bind to specific receptors on immune cell surfaces and alter cell function. They serve to maintain brain homeostasis in the event of illness, injury, or other threats to the CNS by first exerting proinflammatory effects on target cells, which signal activation and ready the cells, like microglia, to respond appropriately to the insult. After the threat is properly eliminated, anti-inflammatory cytokines are released by responding to microglia, which, in turn, signal them to return more closely to a resting state.

As aforementioned, however, the microglia are often not successful in completely eliminating the Aβ deposits in AD. Subsequent cytokine signaling, though aiming to aid in activating microglia, may actually serve to increase amyloidosis. Take the pleiotropic cytokine interleukin-6 as an example. Interleukin-6 (IL-6) has important implications for immune response; it can serve as both an inflammatory cytokine in response to immune threats and exert anti-inflammatory effects after the injury or infection is resolved. Researchers have observed IL-6 in areas of the brain laden with Aβ plaque; most notably, it can be detected in such plaque before pathological changes in the surrounding neurons occur [15]. This fact suggests that IL-6 may play a causative role in the increase in amyloid plaque formation and subsequent neuronal damage. This cytokine, along with tumor necrosis factor-alpha (TNF-a), was shown to increase the activity of the β-site amyloid precursor protein (APP)-cleaving enzyme (BACE1), as well as nuclear factor κB (NFκB), which are directly responsible for the formation of Aβ [16]. This, in turn, creates a vicious, positive feedback loop as more microglia are activated in response, resulting in an upregulation of IL-6, the further formation of Aβ fibrils, deleterious neuritic changes, and synaptic degradation.

Another example of potentially deleterious cytokine signaling can be found in TNF-a pathways. In the healthy brain, TNF-a is found in very low concentrations, but it is upregulated in the brains of AD patients due largely to microglial activation via the TNF receptors (TNFRs) [17]. In non-pathological settings, modest levels of TNF-a are expressed in response to threats such as pathogens or tumor cell growth and serve to activate and direct microglia to eliminate the threat via proinflammatory pathways of the TNFR1. Once the threat is eliminated, TNFR2 pathways serve anti-inflammatory purposes and help restore homeostasis [18]. On its own, TNF-a does not appear to pose a threat to surrounding neurons, but rather, it serves as a protective signaler that alerts microglia of potential danger. In cultures, the presence of elevated TNF-a alone does not result in neuronal loss [17]. Only when elevated TNF-a is in the presence of microglia does the subsequent death of surrounding neurons occur. Most importantly, a single dose of the high-concentration TNF-a solution was not sufficient to induce neuronal loss. Rather, repeated doses were necessary to trigger neuronal phagocytosis. The constant exposure of microglia to Aβ in the AD model likely recreates these effects and causes a prolonged dysregulation in TNF-a signaling, which leads to hallmark neuronal loss [17].

An example of these effects can be observed in the choroid plexus (CP) region of the brain. The CP is composed of tight junction-forming endothelial cells that make up the blood–cerebrospinal fluid barrier (BCSFB) and is an important mediator of cerebrospinal fluid secretion and growth factor production. Additionally, the CP monitors inflammatory markers throughout the body and is responsible for trafficking peripheral immune cells into the brain across the BCSFB [19]. In the AD mouse model, TNF-a was found to be the most upregulated cytokine in the CP, where it leads to diffuse inflammation, microglial activation, and the subsequent degradation of the epithelial cell structure. Indeed, researchers observed noticeably altered epithelial cell shapes and increased MMP-3 production in the CP of those with Aβ deposits. The altered cell shape, when coupled with an increase in matrix-degrading MMP-3, subsequently leads to the breakdown of tight-junction proteins and increases the permeability of the BCSFB [18]. The upregulation of proinflammatory cytokines, like TNF-a, recruits more circulating immune cells, such as leukocytes, and the compromised selective permeability of the CP barrier allows for a greater number of leukocytes to more easily cross from the periphery to the brain parenchyma, thus increasing overall inflammation in the CNS [20]. In this manner, the toxicity of amyloid deposits is increased in this region, and subsequent degradation of the BCSFB only leads to greater levels of proinflammatory markers, both locally and those recruited from the periphery. In turn, increased microglial activation perpetuates this loop, resulting in a prolonged inflammatory state and eventual cell death. Interestingly, when a TNFR blocker was introduced in both in vitro and in vivo settings, microglial-induced neuronal loss was halted and further prevented, indicating that TNFR1 may be an important therapeutic target for reducing overall amyloid toxicity [21]. Moreover, in mice with blocked TNF1 receptors, epithelial cell morphology was preserved in the CP, and a notable reduction in CP and hippocampal inflammatory markers was observed [19]. Perhaps most importantly, cognitive impairments were reduced, and researchers noted improvements in working memory compared to the mice in which TNFR1 had not been abrogated. These results highlight the promise of TNFR1 as a potential target for new drug therapies as a means of reducing overall amyloid toxicity through the curtailment of prolonged inflammatory responses.

### 3.3. Peripheral Inflammation

While the role of neuroinflammation in AD has garnered much more attention in recent years, peripheral inflammatory events in AD pathology also deserve careful research. For example, a longitudinal study published in 2019 by Walker et al. examined how inflammatory markers present during a person’s midlife related to cognitive changes over the course of a 20-year period [22]. The researchers generated a midlife inflammation composite score for each participant, which included measures of such biomarkers as fibrinogen, leukocyte count, and C-reactive proteins (CRPs), as well as a cognitive composite score composed of memory, executive functions, and language abilities. For each standard deviation (SD) increase in the inflammation composite score, participants’ cognitive composite scores decreased by 0.035 SD at the 20-year mark. Moreover, this effect seemed to have a dose-dependent response, with subjects in the highest quartile of inflammatory composite scores showing nearly an 8% steeper decline than those in the lowest quartile. Specifically, participants with CRP in the highest quartile showed a decline that was nearly 12% steeper than those in the lowest quartile. While a one-time finding of elevated CRP alone might not account for these findings, it may suggest that prolonged, elevated CRP could play an important role in activated microglial responses, but this hypothesis needs to be further explored. As previously noted, this type of prolonged activation contributes to synaptic loss through phagocytic processes [22]. These results seem to indicate that systemic inflammatory factors during midlife may play an important role in facilitating cognitive decline later in life.

Indeed, several studies have identified systemic inflammatory conditions that are associated with a higher risk of developing AD later in life. Rheumatoid arthritis (RA), for example, is an autoimmune condition associated with increased peripheral inflammation and inflammatory markers such as the cytokine TNF-a, which is also upregulated in the brains of AD patients. A recent population study determined that RA is a major risk factor for the subsequent development of AD [23]. Anti-TNF therapies are popular treatments for RA, and recent findings indicate that controlling this inflammatory signaler can significantly reduce dementia risk [24].

Even certain metabolic syndromes that are associated with inflammation, such as atherosclerosis and obesity, correlate to increased risk for the development of AD later in life. These conditions are associated with excessive nutrient intake, often attributed to the “Western diet”. In a rodent model, a high-fat diet was associated with the increased secretion of proinflammatory cytokines and subsequent inflammation in the hippocampus, reduced neurogenesis, and cognitive decline due to microglial activation [25]. Diabetes mellitus (DM), a common metabolic condition among US adults, has long been known to increase risk factors for vascular conditions, but recent evidence has linked it to increased risk for AD as well. This association may be due, in part, to peripheral inflammatory markers characteristic of DM that exert an effect on the CNS and play a role in the pathomechanism of AD. Specifically, nuclear factor-kappa B (NFkB) is upregulated in diabetics as a result of either hyperglycemic conditions or inflammatory cytokine signaling related to the increased adiposity commonly associated with DM [26]. NFkB can contribute indirectly to neuroinflammation by aiding in the formation of Aβ; this effect is achieved by binding to the promotor of BACE1, which, in turn, causes an increased expression of β-secretase, resulting in the production of amyloid fibrils and increases the risk of developing AD [27]. Moreover, when activated (such as in diabetic persons), NFkB exerts influence on genes that encode cytokines and chemokines, causing a secondary inflammatory cascade [28]. Because NFkB contributes to the upregulation of proinflammatory cytokines and is simultaneously activated by said cytokines, a self-maintaining feedback loop is created, which increases inflammatory processes and contributes to the maintenance of chronic inflammation in both the periphery and CNS [29]. Not surprisingly, this cycle results in worse cognitive outcomes over time. In a study of over 1000 adults with type-2 DM, researchers observed elevated CRP, IL-6, and TNF-a plasma levels when compared to healthy adults based on population data. Even after controlling for age and sex, higher levels of circulating inflammatory markers in those with type-2 DM correlated strongly and negatively with cognitive performance [30].

Moreover, DM is associated with advanced glycation end (AGE) products, which are formed by the non-enzymatic reaction of glucose and other saccharides with proteins, lipids, and nucleotides. Receptors for advanced glycation end products (RAGEs) are found in peripheral nerves, where they interact with AGE and may lead to inflammation and apoptosis. In fact, the concentration of RAGEs is elevated in nerve cells affected by diabetic neuropathy [31]. Additionally, AGE products can lead to an upregulation of other inflammatory cytokines, such as TNF-a and IL-1 beta, as well as NFkB, which mediates inflammation and apoptosis. Insulin resistance and AGE products have also been implicated in the development of AD and other types of dementia, where impaired glucose processing leads to mitochondria dysfunction and, ultimately, synaptic dysfunction.

Finally, peripheral inflammatory events such as bacterial infection may pose a risk for the development of AD. A systematic review analyzed outcomes of over 890,000 subjects and found that sepsis survivors were at significantly higher risk of developing dementia [32]. To determine the mechanism of these findings, researchers induced sepsis in an AD rodent model. They observed an increase in Aβ formation and elevated levels of immune response-related ligands called receptors for advanced glycation end products (RAGEs), which are capable of activating inflammatory pathways. The infection also compromised blood–brain barrier (BBB) permeability, allowing peripheral inflammatory markers such as IL-6 and TNF-a to enter the brain. This, in turn, activated resident microglia, leading to an increased neuroinflammatory response along with impaired cognitive performance. Interestingly, when RAGE antibodies were introduced into the prefrontal cortex, and hippocampus, Aβ deposition, and microglial activation were inhibited, thus highlighting RAGE as a potential therapeutic target for preventing AD, especially in post-sepsis older adults [33]. These results support the idea that comorbid inflammatory conditions, both mild and severe, contribute to neuroinflammation and subsequent cognitive decline in AD.

## 4. Discussion

Neuroinflammation can no longer be considered a symptom of AD but rather a driving force in the onset and progression of the disease. Characterized by the chronic, prolonged activation of microglia via proinflammatory cytokine signaling pathways, neuroinflammation is a protective response to Aβ deposition gone awry. Rather than aiding in the clearance of Aβ, it serves to increase overall amyloid toxicity through synaptic degradation, the production of membrane-degrading compounds, the recruitment of peripheral immune cells through compromised barriers, and ultimately, further Aβ accumulation. Based on the knowledge of the role of inflammation in AD progression, a number of studies evaluated the potential role of NSAID treatments in the prevention and treatment of AD. Unfortunately, none of these approaches showed efficacy, with many associated with adverse events of different severities [34,35]. The question remains of how both inflammatory processes and potent anti-inflammatory treatments coexist. We hypothesize that the relationships between AD and different elements of inflammatory processes are complex and most likely imply the dysregulation of different inflammatory patterns that facilitate the formation of the amyloid in the early stages and the propagation of tau in the later stages of AD; these processes also impair the ability of microglia to phagocytize AB42. Specifically, in a recent study, certain leukocyte surface biomarkers showed profound dysregulation in the early stages of amyloid accumulation, again supporting the hypothesis that the dysfunction, rather than an over-response of the immune system, is responsible for facilitating the biological processes of AD [36]. As we move to explore future AD-specific immunotherapies, we believe that the target selection should aim to address the process leading to the dysregulation of the immune system and reestablish appropriate functioning rather than suppressing the immune response, which can perhaps result in further neuronal damage. While there is a myriad of investigational drugs in the research pipeline that show promise in modulating these inflammatory processes, more research is needed into treatments of neuroinflammation [37]. Promising therapies will more than likely include combination approaches aimed at reducing or altogether preventing chronic microglial activation via halting multiple cytokine cascades at the receptor level, restoring compromised BCSFB and BBB, and reducing peripheral inflammatory markers.

## 5. Conclusions

The emerging literature clearly shows that neuroinflammation plays a significant role in the pathology of AD. Further understanding of the specific roles of inflammatory biomarkers will undoubtedly illuminate new targets for therapeutic development and provide a better understanding of the disease process.

### Future Directions

The availability of new imaging, as well as biomarker technology, has given us new insights into our understanding of AD. Today, we know that not all individuals with amyloid pathology will develop clinical AD, and not all individuals with clinical AD will present an amyloid pathology. Furthermore, new research shows that most individuals with clinical features of AD will present with numerous pathological processes that indicate the presence of a debilitated brain unable to overcome neurodegeneration. The field of neuroinflammation as it relates to AD is rapidly evolving. As we seek to better understand the immune factors that contribute to AD’s pathology, we hope that some of the previously formulated questions will be answered and new targets for therapeutic development will be identified. We expect that immune therapeutic targets will become a key component in the treatment and, eventually, the cure of AD.

## Data Availability

No new data were created or analyzed in this study. Data sharing is not applicable to this article.
